# M-Polynomials and topological indices of V-Phenylenic Nanotubes and Nanotori

**DOI:** 10.1038/s41598-017-08309-y

**Published:** 2017-08-18

**Authors:** Young Chel Kwun, Mobeen Munir, Waqas Nazeer, Shazia Rafique, Shin Min Kang

**Affiliations:** 1 Department of Mathematics, Dong-A University, Busan, 49315 Korea; 2 Division of Science and Technology, University of Education, Lahore, 54000 Pakistan; 3 Center for Excellence in Molecular Biology, Punjab University Lahore, Lahore, 53700 Pakistan; 4 Department of Mathematics and Research Institute of Natural Science, Gyeongsang National University, Jinju, 52828 Korea; 5 Center for General Education, China Medical University, Taichung, 40402 Taiwan

## Abstract

V-Phenylenic nanotubes and nanotori are most comprehensively studied nanostructures due to widespread applications in the production of catalytic, gas-sensing and corrosion-resistant materials. Representing chemical compounds with M-polynomial is a recent idea and it produces nice formulas of degree-based topological indices which correlate chemical properties of the material under investigation. These indices are used in the development of quantitative structure-activity relationships (QSARs) in which the biological activity and other properties of molecules like boiling point, stability, strain energy etc. are *correlated* with their structures. In this paper, we determine general closed formulae for M-polynomials of V-Phylenic nanotubes and nanotori. We recover important topological degree-based indices. We also give different graphs of topological indices and their relations with the parameters of structures.

## Introduction

Mathematical models, based on polynomial-representations of chemical compounds, can be used to predict their properties. Mathematical chemistry is rich in tools such as polynomials and functions which can forecast properties of compounds. Topological indices are numerical parameters of a graph which characterize its topology and are usually graph invariant. They describe the structure of molecules numerically and are used in the development of quantitative structure activity relationships (QSARs). A degree-based topological index is one sub-class where index is computed on the basis of degrees of molecular graph. These numerical values correlate structural facts and chemical reactivity, biological activities and physical properties^1–5^. Experiments and results revealed that material properties such as boiling point, strain energy, heats of formation and reaction, viscosity, radius of gyration, and fracture toughness of a molecule are tightly connected to its structure and this fact plays a dominant role in chemical graph theory^1, 3, 6–8^.

Usually topological indices are computed using definitions^9–15^. One wishes to find a compact general method that can produce many topological indices of a certain category^16, 17^. One well-established method is the computation of a general polynomial whose derivatives or integrals or blend of both, evaluated at some particular point yield topological indices. For example, Hosoya polynomial (also called Wiener polynomial), is a general polynomial whose derivatives evaluated at 1 produce Weiner and Hyper Weiner indices^16^. This polynomial is considered to be the most general polynomial in the context of determination of distance-based topological indices. Thus computations of distance-based topological indices reduce to computation of one single polynomial.

The M-polynomial plays parallel role in the context of degree-based topological indices and give closed formulas of more than ten degree-based topological indices^17–22^. It is the most general polynomial developed up till now. This, in particular, implies that knowing the M-polynomial of a given family of graphs, a closed formula for any such index can be obtained routinely. Moreover, we hope that a deeper analysis of the properties of the M-polynomial will open up new general insights in the study of degree-based topological indices. Although there are other polynomials like Zagreb polynomials and Forgotten polynomial but these polynomials only give one or two degree-based topological indices (for details see^23, 24^).

Rapid advancements are being made in this field on day to day basis. In this report, we intend to compute the M-Polynomials and Topological indices of V-Phenylenic nanotubes and nanotori. The structures of V-Phenylenic nanotubes and nanotorus consist of several *C*_4_ *C*_6_ *C*_8_ nets. A *C*_4_ *C*_6_ *C*_8_ net is a trivalent decoration made by alternate *C*_*4*_, *C*_*6*_, and *C*_*8*_. Phenylenes are polycyclic conjugated molecules, composed of four-membered and six-membered rings such that every four-membered ring is adjacent to two six-membered rings and a four-membered ring is adjacent to two eight-membered rings. We denote the V-Phenylenic nanotubes and nanotorus by *VPHX*[*m*, *n*], and *VPHY*[*m*, *n*] respectively, where m and n are the number of atoms in rows and columns. In ^9^, authors computed Pi polynomial and related topological indices of V-Phenylenic nanotubes and nanotori and in^10^ computed general forms of theta polynomial and theta index of these materials. Many well-known topological indices of these materials have been recently computed. For example, authors computed vertex Pi index in^11^, *GA* and atom-bond connectivity index in^12^, eccentricity connectivity index in^13^, *GA*_*5*_ index in^14^ and fourth atom-bond connectivity index in^15^.

All graphs in this report are connected and simple. For the rest of this article we reserve V(*G*) *as set of vertices*, *E*(*G*) as the set of edges and *d*_*v*_ as the degree of a vertex *v*.

**Definition 1**. The M-polynomial associated to a graph G denoted as *M* (*G*, *x*, *y*) is^17^

1 $$M(G,x,y)=\sum _{\delta \le i\le j\le {\rm{\Delta }}}{m}_{ij}(G){x}^{i}{y}^{j},$$

where *δ* = *Min*{*d*_*v*_|*v* ∈ *V* (*G*)},Δ = *Max*{*d*_*v*_|*v* ∈ *V* (*G*)}, and *m*_*ij*_(*G*) is the edge *vu* ∈ *E*(*G*) such that {*d*_*v*_, *d*_*u*_} = {*i*, *j*}.

**Figure 1:**
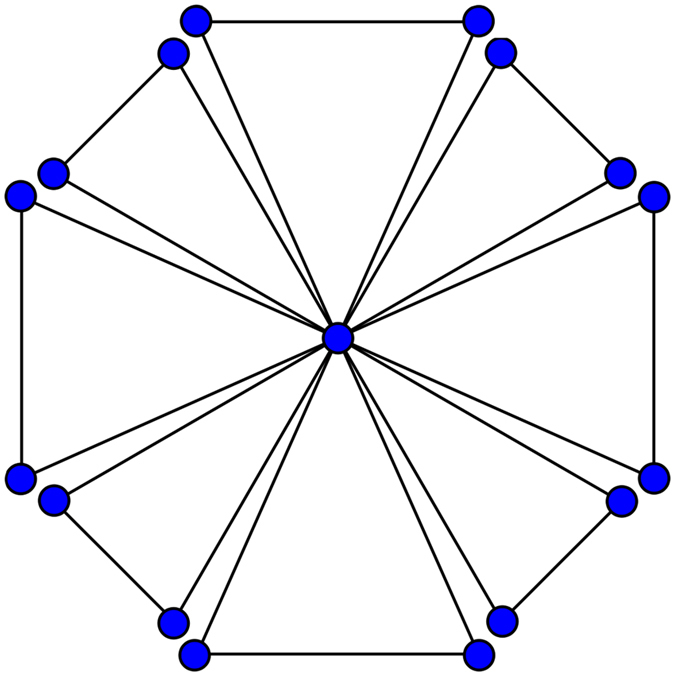
Graph G.

Example: Let we have a graph *G* shown in Fig. 1 for which |*E*(*G*)| = 24 and |*V*(*G*)| = 17. It is easy to see that the set of vertices of *G* can be divided into two parts with respect to degree of the vertices, i.e. *V*_1_(*G*) = {*v* ∈ *V*(*G*): *d*_*v*_ = 2} and *V*_2_(*G*) = {*v* ∈ *V*(*G*): *d*_*v*_ = 16} such that |*V*_1_(*G*)| = 16 and |*V*_2_(*G*)| = 1. The set of edges of *G* also has two partitions, i.e. *E*_1_(*G*) = {*uv* ∈ *E*(*G*): d_*u*_ = *d*_*v*_ = 2} and *E*_2_(*G*) = {*uv* ∈ *E*(*G*): d_*u*_ = 2 and *d*_*v*_ = 16} such that |*E*_1_(*G*)| = 8 and |*E*_2_(*G*)| = 16. Also we can observe that *δ* = 2 and Δ = 16.

Hence by (1) we get

$$\begin{array}{rcl}M(G;x,y) & = & \sum _{2\le i\le j\le 16}\,{m}_{ij}{x}^{i}{y}^{j}\\  & = & \sum _{2\le 2}{m}_{22}{x}^{2}{y}^{2}+\sum _{2\le 16}{m}_{216}{x}^{2}{y}^{16}\\  & = & \sum _{uv\in {E}_{1}(G)}{m}_{22}{x}^{2}{y}^{2}+\sum _{uv\in {E}_{2}(G)}{m}_{216}{x}^{2}{y}^{16}\\  & = & |{E}_{1}(G)|{x}^{2}{y}^{2}+|{E}_{2}(G)|{x}^{2}{y}^{16}\\  & = & 8{x}^{2}{y}^{2}+16{x}^{2}{y}^{16}.\end{array}$$

Path number was the first distance-based topological index formulated by Wiener^25^, now known as *Wiener index*. Gradually it became famous because of its various applications; see for details^6, 26^. Later Milan Randić in 1975, introduced Randić index^27^, *R*_−1/2_(*G*), given by

2 $${R}_{-1/2}(G)=\sum _{uv\in E(G)}\frac{1}{\sqrt{{d}_{u}{d}_{v}}}.$$

The generalized Randić index was proposed independently by Bollobas *et al*.^28^ and Amic *et al*.^29^ in 1998 and has been studied extensively by both chemist and mathematicians^30^. A detailed survey is given in ref. 31.

The general version of Randić index is

3 $${R}_{\alpha }(G)=\sum _{uv\in E(G)}\frac{1}{{({d}_{u}{d}_{v})}^{\alpha }},$$

and the inverse Randić index is defined as $R{R}_{\alpha }(G)=\sum _{uv\in E(G)}{({d}_{u}{d}_{v})}^{\alpha }$.

Obviously for $\alpha =-\frac{1}{2}$ in (3), we obtain *R*_−1/2_(*G*) as its particular case.

The Randić index has lots of applications in many diverse areas^32–38^ including drug designs. There are many reasonable arguments about the physical usage of such a simple graph invariant, but the actual fact is still a mystery.

First and second Zagreb indices^23, 24, 39^, were introduced by Gutman and Trinajstić defined as: ${M}_{1}(G)=$ $\sum _{uv\in E(G)}({d}_{u}+{d}_{v})$ and ${M}_{2}(G)=\sum _{uv\in E(G)}({d}_{u}\times {d}_{v})$ respectively whereas second modified Zagreb index is:

4 $${}^{m}M_{2}(G)=\sum _{uv\in E(G)}\frac{1}{{d}_{u}{d}_{v}}.$$

The Symmetric division index which determines surface area of polychlorobiphenyls is defined as:

5 $${\rm{S}}{\rm{D}}{\rm{D}}({\rm{G}})=\sum _{uv\in E(G)}\{\frac{min({d}_{u},{d}_{v})}{max({d}_{u},{d}_{v})}+\frac{max({d}_{u},{d}_{v})}{min({d}_{u},{d}_{v})}\}.$$

The other version of Randic index is harmonic index defined as:

6 $$H(G)=\sum _{vu\in E(G)}\frac{2}{{d}_{u}+{d}_{v}}.$$

*I*(*G*), Inverse sum-index, is

7 $$I(G)=\sum _{vu\in E(G)}\frac{{d}_{u}{d}_{v}}{{d}_{u}+{d}_{v}}.$$

*A*(*G*), augmented Zagreb index, is

8 $$A(G)=\sum _{vu\in E(G)}{\{\frac{{d}_{u}{d}_{v}}{{d}_{u}+{d}_{v}-2}\}}^{3}.$$

This index gives best approximation of heat of formation of alkanes^40, 41^. Let *M*(*G*; *x*, *y*) = *f*(*x*, *y*) then the following Table 1 relates above described topological indices with M-polynomial^17^.Where

$$\begin{array}{ccc}{Q}_{\alpha }(f(x,y)) & = & {x}^{\alpha }f(x,y),\quad {D}_{x}=x\frac{{\rm{\partial }}(f(x,y))}{{\rm{\partial }}x},\\ {D}_{y} & = & \,y\frac{{\rm{\partial }}(f(x,y))}{{\rm{\partial }}y},\quad {S}_{x}={\int }_{0}^{x}\frac{f(t,y)}{t}dt,\\ {S}_{y} & = & {\int }_{0}^{y}\frac{f(x,t)}{t}dt,\quad J(f(x,y))=f(x,x).\,\end{array}$$

**Table 1:** Relations of topological indices with M-polynomial.

Topological Index	Derivation from *f*(*x*, *y*)
*M*_1_(*G*)	(*D*_*x*_ + *D*_*y*_)(*f*(*x*, *y*))_*x*=*y*=1_
*M*_2_(*G*)	(*D*_*x*_ *D*_*y*_)(*f*(*x*, *y*))_*x*=*y*=1_
^*m*^ *M*_2_(*G*)	(*S*_*x*_ *S*_*y*_)(*f*(*x*, *y*))_*x*=*y*=1_
*RR*_*α*_(*G*), *α* ∈ *N*	$({D}_{x}^{\alpha }{D}_{y}^{\alpha }){(f(x,y))}_{x=y=1}$
*R*_*α*_(*G*), *α* ∈ *N*	$({S}_{x}^{\alpha }{S}_{y}^{\alpha }){(f(x,y))}_{x=y=1}$
SDD(G)	(*D*_*x*_ *S*_*y*_ + *S*_*x*_ *D*_*y*_)(*f*(*x*, *y*))_*x*=*y*=1_
*H*(*G*)	2*S*_*x*_ *J*(*f*(*x*, *y*))_*x*=1_
*I*(*G*)	*S*_*x*_ *JD*_*x*_ *D*_*y*_(*f*(*x*, *y*))_*x*=1_
*A*(*G*)	${{S}_{x}}^{3}{Q}_{-2}J{{D}_{x}}^{3}{{D}_{y}}^{3}{(f(x,y))}_{x=1}$

## Main Results

Here we present our main results, starting with the general closed forms of M-polynomials of V-Phenylenic nanotubes *VPHX*[*m*, *n*] with m and n taking only positive integral value. Then we compute M-polynomial of V-Phenylenic nanotori *VPHY*[*m*, *n*]. In the end, we rapidly compute many topological indices from the derived M-polynomials. We also give 3-D Plots of these polynomials using Maple 13 developed by Maplesoft. For convenience, we place V-Phenylenic nanotubes *VPHX*[*m*, *n*] and V-Phenylenic nanotori *VPHY*[*m*, *n*] into two different sections. For the rest of this article, we take *m* and *n* to be positive integers.

### Computational aspects of V-Phynelenic nanotubes

**Figure 2:**
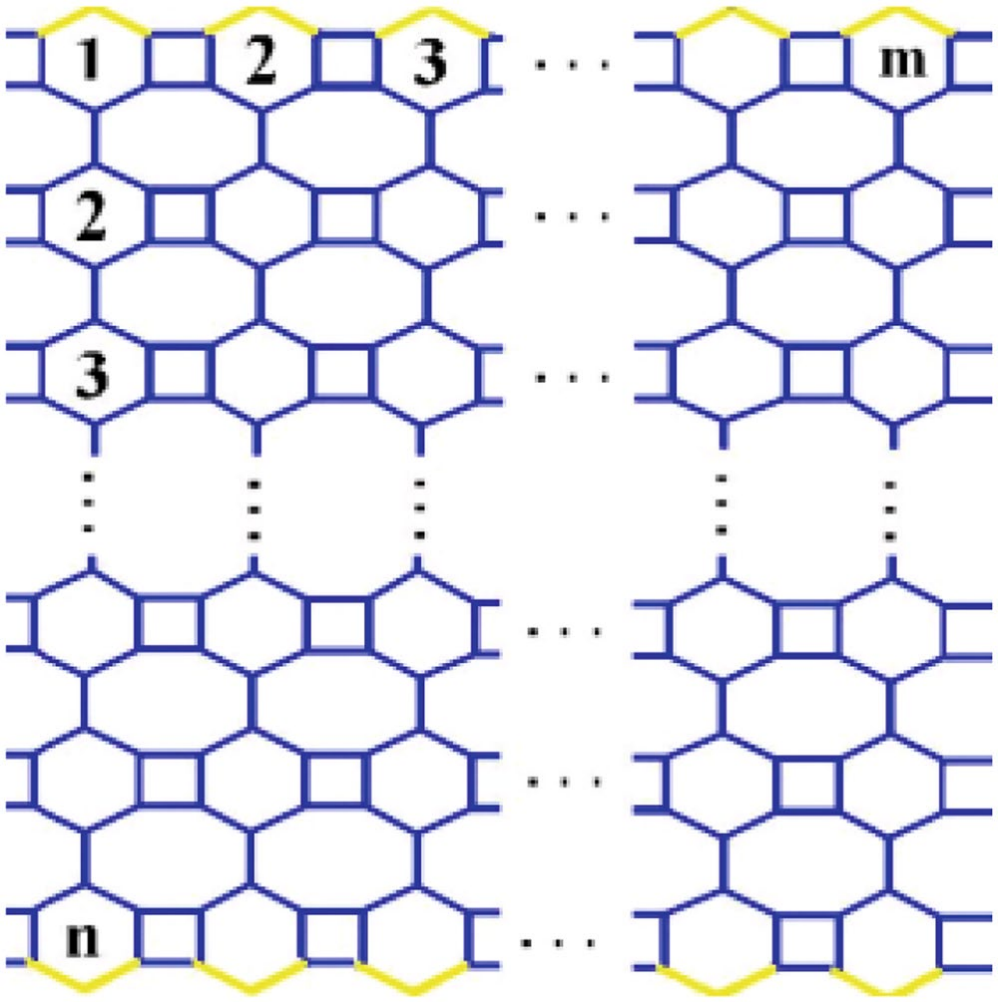
2-D Lattice molecular graph of V-Phynelenic nanotube *VPHX*[*m*, *n*].

Let G=VPHX[m, n] be the V-Phenylenic nanotubes. From Fig. 2, we see that the graph has *6mn* number of vertices and *9mn* number of edges. The vertex partition and edge partition of graph *G* are shown in Tables 1 and 2 respectively.

**Table 2:** The partition of *V*(*G*) of *G* = *VPHX*[*m*, *n*].

*d*_*v*_	*2*	*3*
Number of vertices	*2* *m*	*6* *mn* – *2* *m*

**Theorem 1:** Let *VPHX*[*m*, *n*] is the V-Phenylenic nanotubes then

9 $$M(VPHX[m,n];x,y)=4m{x}^{2}{y}^{3}+m(9n-5){x}^{3}{y}^{3}.$$

**Proof:** Let *VPHX*[*m, n*] is V-Phenylenic nanotubes, where *m* and *n* are the numbers of atoms in each row and column respectively. It is easy to see from Fig. 2 that

10 $$|V(VPHX[m,n])|=6mn,$$

11 $$|E(VPHX[m,n])|=9\,mn\mbox{--}m.$$

From Table 2, the vertex set of *VPHX*[*m*, *n*] have two partitions:

12 $${V}_{1}(VPHX[m,n])=\{u\in V(VPHX[m,n]):{d}_{u}=2\},$$

13 $${V}_{2}(VPHX[m,n])=\{u\in V(VPHX[m,n]):{d}_{u}=3\}$$

such that

14 $$|{V}_{1}(VPHX[m,n])|=2m,$$

15 $$|{V}_{2}(VPHX[m,n])|=6mn-2m.$$

From Table 3, the edge set of *VPHX*[*m*, *n*] have two partitions:

16 $${E}_{1}(VPHX[m,n])=\{e=uv\in E(VPHX[m,n]):{d}_{u}=2,{d}_{v}=3\},$$

17 $${E}_{2}(VPHX[m,n])=\{e=uv\in E(VPHX[m,n]):{d}_{u}={d}_{v}=3\}.$$

**Table 3:** Edge partition of edge sets of *G* = *VPHX*[*m*, *n*].

*(d*_*u*_ *, d*_*v*_ *)*	*(2, 3)*	*(3, 3)*
Number of edges	*4m*	*m(9n* − *5)*

In Fig. 2 yellow labeled edges correspond to *E*_1_ and back labeled edges correspond to *E*_2_.

18 $$|{E}_{1}(VPHX[m,n])|=4m,$$

19 $$|{E}_{2}(VPHX[m,n])|=|E(VPHX[m,n])|-4m=9mn-m-4m=m(9n-5).$$

Now from the definition of the M-polynomial

20 $$\begin{array}{rcl}M(VPHX[m,n];x,y) & = & \sum _{i\le j}{m}_{ij}{x}^{i}{y}^{j}\\  & = & \sum _{2\le 3}{m}_{23}{x}^{2}{y}^{3}+\sum _{3\le 3}{m}_{33}{x}^{3}{y}^{3}\\  & = & \sum _{uv\in {E}_{1}(VPHX[m,n])}{m}_{23}{x}^{2}{y}^{3}+\sum _{uv\in {E}_{2}(VPHX[m,n])}{m}_{33}{x}^{3}{y}^{3}\\  & = & |{E}_{1}(VPHX[m,n])|{x}^{2}{y}^{3}+|{E}_{2}(VPHX[m,n])|{x}^{3}{y}^{3}\\  & = & 4m{x}^{2}{y}^{3}+m(9n-5){x}^{3}{y}^{3}.\end{array}$$

**Figure 3:**
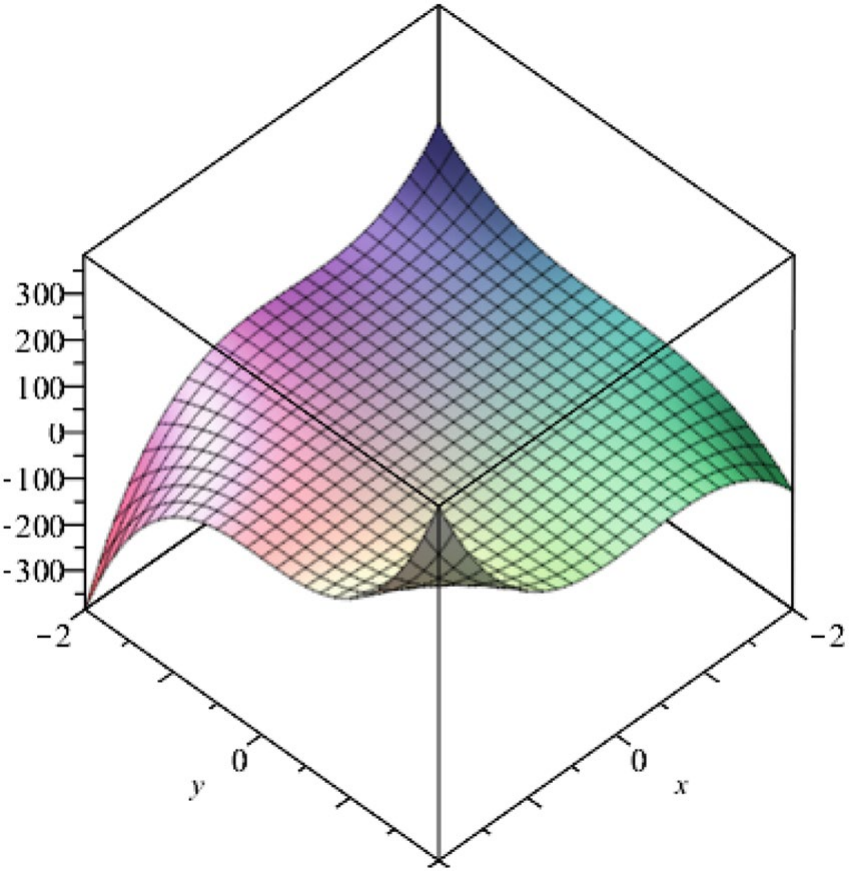
The plot of the M-polynomial of V-Phynelenic nanotube *VPHX*[*1*, *1*].

Above Fig. 3 is plotted using Maple 13. This suggests that values obtained by M-polynomial show different behaviors corresponding to different parameters x and y. We can control values of M-polynomial through these parameters. Clearly, Fig. 2 shows that along one side intercept is an upward opening parabola and along the other side is the downward parabola.

Following proposition computes topological indices of V-Phenylenic nanotubes.

**Proposition 2**. Let *VPHX*[*m*, *n*] is the V-Phenylenic nanotubes.

1. *M*_1_(*VPHX*[*m*, *n*]) = 20*m* + 6*m*(9*n* − 5).
2. *M*_2_(*VPHX*[*m*, *n*]) = 24*m* + 9*m*(9*n* − 5).
3. $${}^{m}M_{2}(VPHX[m,n])=\frac{2}{3}m+\frac{1}{9}m(9n-5).$$
4. *R*_*α*_(*VPHX*[*m*, *n*]) = 2^*α*^+ ^2^3^*α*^ *m* + 3^2*α*^ *m*(9*n* − 5).
5. $$R{R}_{\alpha }(VPHX[m,n])=\frac{m}{{2}^{\alpha -2}{3}^{\alpha }}+\frac{m(9n-5)}{{3}^{2\alpha }}.$$
6. $$SSD(VPHX[m,n])=\frac{26}{3}m+2m(9n-5).$$
7. $$H(VPHX[m,n])=\frac{4}{5}m+\frac{1}{6}m(9n-5).$$
8. $$I(VPHX[m,n])=\frac{24}{5}m+\frac{3}{2}m(9n-5){\rm{.}}$$
9. $$A(VPHX[m,n])=32m+\frac{729}{64}m(9n-5).$$

**Proof**.

Let

21 $$M(VPHX[m,n];x,y)=f(x,y)=4m{x}^{2}{y}^{3}+m(9n-5){x}^{3}{y}^{3}.$$

Then

22 $${D}_{x}(f(x,y))=8m{x}^{2}{y}^{3}+3m(9n-5){x}^{3}{y}^{3},$$

23 $${D}_{y}(f(x,y))=12m{x}^{2}{y}^{3}+3m(9n-5){x}^{3}{y}^{3},$$

24 $$({D}_{y}{D}_{x})(f(x,y))=24m{x}^{2}{y}^{3}+9m(9n-5){x}^{3}{y}^{3},$$

25 $${S}_{x}{S}_{y}(f(x,y))=\frac{2}{3}m{x}^{2}{y}^{3}+\frac{1}{9}m(9n-5){x}^{3}{y}^{3},$$

26 $${{D}_{x}}^{\alpha }{{D}_{y}}^{\alpha }(f(x,y))={2}^{\alpha +2}\times {3}^{\alpha }m{x}^{2}{y}^{3}+{3}^{2\alpha }m(9n-5){x}^{3}{y}^{3},$$

27 $${{S}_{x}}^{\alpha }{{S}_{y}}^{\alpha }(f(x,y))=\frac{1}{{2}^{\alpha -2}{3}^{\alpha }}m{x}^{2}{y}^{3}+\frac{1}{{3}^{2\alpha }}m(9n-5){x}^{3}{y}^{3},$$

28 $${S}_{y}{D}_{x}(f(x,y))=\frac{8}{3}m{x}^{2}{y}^{3}+m(9n-5){x}^{3}{y}^{3},$$

29 $${S}_{x}{D}_{y}(f(x,y))=6m{x}^{2}{y}^{3}+m(9n-5){x}^{3}{y}^{3},$$

30 $${S}_{x}Jf(x,y)=\frac{4}{5}m{x}^{5}+\frac{1}{6}m(9n-5){x}^{6},$$

31 $${S}_{x}J{D}_{x}{D}_{y}(f(x,y))=\frac{24}{5}m{x}^{5}+\frac{3}{2}m(9n-5){x}^{6},$$

32 $${{S}_{x}}^{3}{Q}_{-2}J{{D}_{x}}^{3}{{D}_{y}}^{3}f(x,y)={2}^{5}m{x}^{3}+\frac{{3}^{6}}{{4}^{3}}m(9n-5){x}^{4}.$$

Now from Table 1

1. $${M}_{1}(VPHX[m,n])={({D}_{x}+{D}_{y})(f(x,y))|}_{x=y=1}=20m+6m(9n-5).$$
2. $${M}_{2}(VPHX[m,n])={{D}_{x}{D}_{y}(f(x,y))|}_{x=y=1}=24m+9m(9n-5).$$
3. $${}^{m}M_{2}(VPHX[m,n])={{S}_{x}{S}_{y}(f(x,y))|}_{x=y=1}=\frac{2}{3}m+\frac{1}{9}m(9n-5).$$
4. *R*_*α*_(*VPHX*[*m*, *n*]) = 2^*α*+2^3^*α*^ *m* + 3^2*α*^ *m*(9*n* − 5).
5. $$R{R}_{\alpha }(VPHX[m,n])=\frac{m}{{2}^{\alpha -2}{3}^{\alpha }}+\frac{m(9n-5)}{{3}^{2\alpha }}.$$
6. $$SSD(VPHX[m,n])={({S}_{y}{D}_{x}+{S}_{x}{D}_{y})(f(x,y))|}_{x=y=1}=\frac{26}{3}m+2m(9n-5).$$
7. $$H(VPHX[m,n])=2{S}_{x}J(f(x,y)){|}_{x=1}=\frac{4}{5}m+\frac{1}{6}m(9n-5).$$
8. $$I(VPHX[m,n])={S}_{x}J{D}_{x}{D}_{y}{(f(x,y))}_{x=1}=\frac{24}{5}m+\frac{3}{2}m(9n-5).$$
9. $$A(VPHX[m,n])={{{S}_{x}}^{3}{Q}_{-2}J{{D}_{x}}^{3}{{D}_{y}}^{3}(f(x,y))|}_{x=1}=32m+\frac{729}{64}m(9n-5).$$

### Computational aspects of the V-Phenylenic nanotori

**Figure 4:**
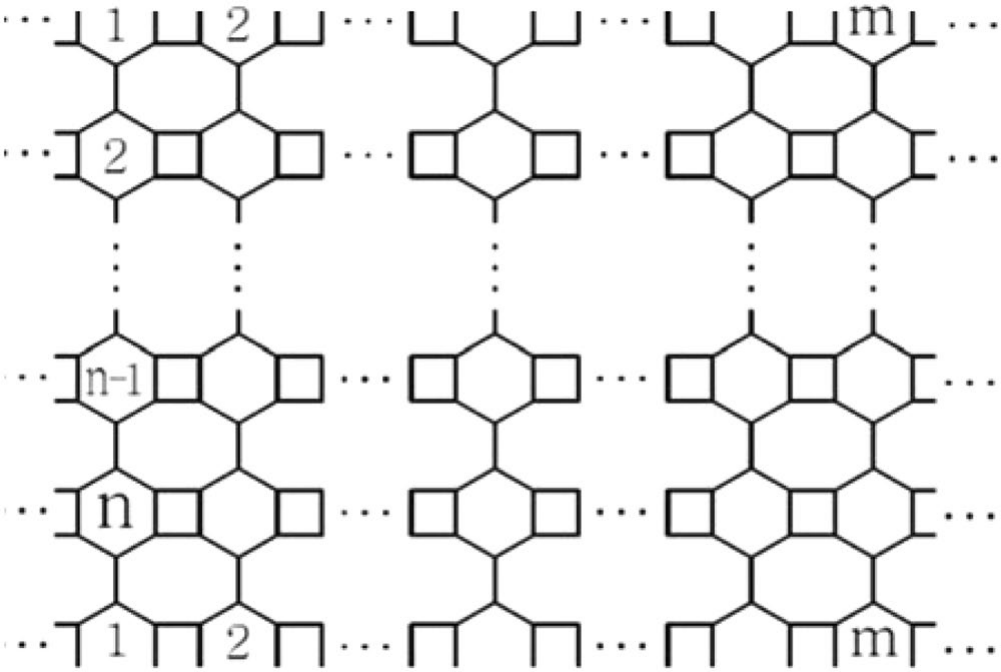
2D-lattice of V-Phenylenic nanotori *VPHY*[*m*, *n*].

Let *VPHY*[*m*, *n*] be the V-Phenylenic nanotori. From Fig. 4, we see that the graph has *6 mn* number of vertices and *9* *mn* edges. The graph has no vertex partitions because every vertex in the graph has degree *3*.

**Theorem 1:** Let *VPHY*[*m*, *n*] is the V-Phenylenic nanotori then

33 $$M(VPHY[m,n];x,y)=9mn{x}^{3}{y}^{3}.$$

**Proof:** Let *VPHY*[*m*, *n*] is V-Phenylenic nanotori, where m and *n* are the number of atoms in each row and column respectively. It is easy to see from Fig. 4 that

34 $$|V(VPHY[m,n])|=6mn,$$

35 $$|E(VPHY[m,n])|=9mn.$$

There is only one type of vertices in the vertex set of *VPHY*[*m*, *n*]:

36 $${V}_{1}(VPHY[m,n])=\{u\in V(VPHY[m,n]):{d}_{u}=3\}$$

such that

37 $$|{V}_{1}(VPHY[m,n])|=6mn.$$

Also the edge set of *VPHY*[*m*, *n*] have only one type of edges:

38 $${E}_{1}(VPHY[m,n])=\{e=uv\in E(VPHY[m,n]):{d}_{u}={d}_{v}=3\}$$

such that

39 $$|{E}_{1}(VPHY[m,n])|=9mn.$$

Now again from (1) we have

40 $$\begin{array}{rcl}M(VPHY[m,n];x,y) & = & \sum _{i\le j}{m}_{ij}{x}^{i}{y}^{j}=\sum _{3\le 3}{m}_{33}{x}^{3}{y}^{3}\\  & = & \sum _{uv\in {E}_{2}(VPHY[m,n])}{m}_{33}{x}^{3}{y}^{3}\\  & = & |{E}_{2}(VPHX[m,n])|{x}^{3}{y}^{3}=9mn{x}^{3}{y}^{3}.\end{array}$$

**Figure 5:**
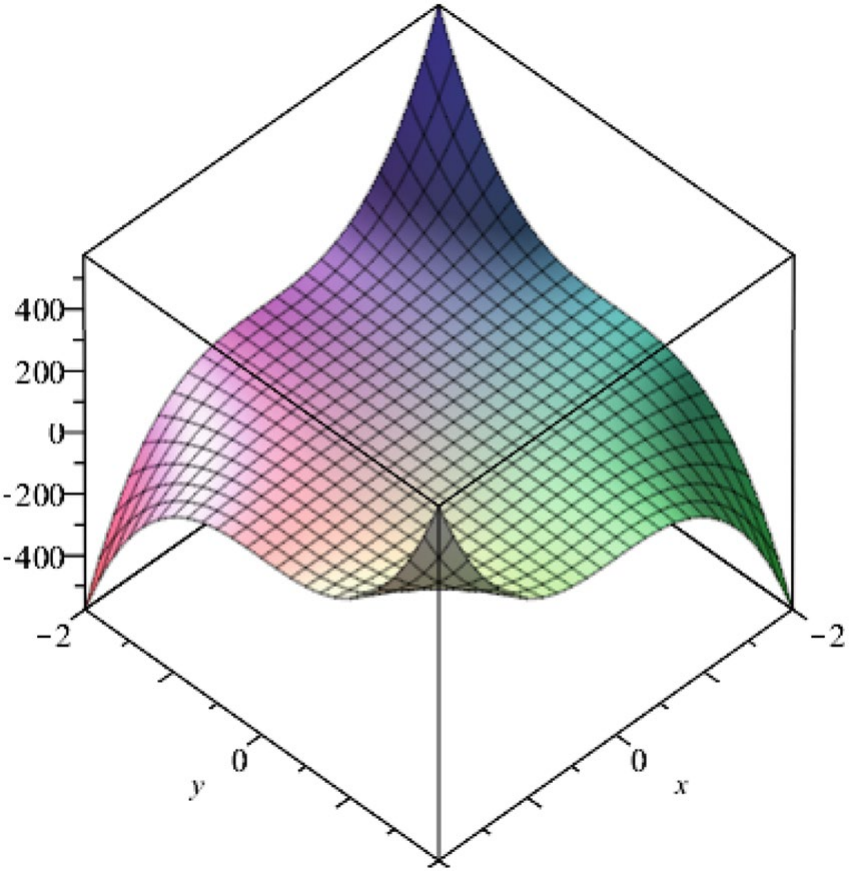
Plot of the M-polynomial of V-Phenylenic nanotori *VPHY*[1, 1].

Above Fig. 5 is plotted using Maple 13. This suggests that values obtained by M-polynomial show different behaviors corresponding to different parameters *x* and *y*. We can control these values through these parameters. Following proposition computes topological indices of V-Phenylenic nanotubes.

**Proposition 2**. Let *VPHY*[*m*, *n*] is the V-Phenylenic nanotori. Then

1. *M*_1_(*VPHY*[*m*, *n*]) = 54*mn*.
2. *M*_2_(*VPHy*[*m*, *n*]) = 81*mn*.
3. ^*m*^ *M*_2_(*VPHY*[*m*, *n*]) = *mn*.
4. *RR*_*α*_(*VPHY*[*m*, *n*]) = 3^2*α*+2^ *mn*.
5. *R*_*α*_(*VPHY*[*m*, *n*]) = 3^2−2*α*^ *mn*.
6. *SSD*(*VPHY*[*m*, *n*]) = 18*mn*.
7. $$H(VPHY[m,n])=\frac{3}{2}mn.$$
8. $$I(VPHY[m,n])=\frac{27}{2}mn.$$
9. $$A(VPHY[m,n])=\frac{{3}^{8}}{{4}^{3}}mn.$$

## Conclusions and Discussions

**Figure 6:**
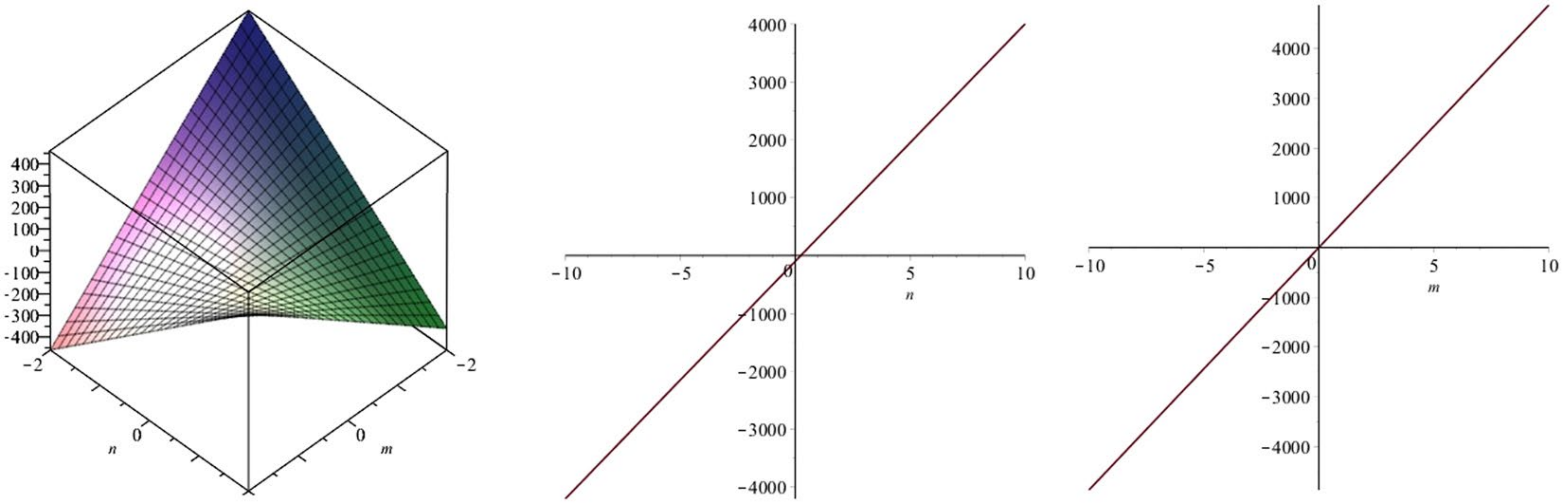
Plots of augmented Zagreb index of *VPHX*[*m*, *n*] 3D left, for *m* = *4* middle and for *n* = *5* right.

We computed general forms of M-polynomials of V-Phenylenic nanotube and nanotori for the first time. Then we computed formulas for many degree-based topological indices with these polynomials. These indices are functions depending upon parameters of structures and are experimentally correlated with many properties. Moreover, we want to conclude that all indices are linearly related with the structural parameters *m* and *n* as the following Fig. 6 suggest. First one is the graph of augmented Zagreb index of V-Phenylenic Nanotube, $A({Z}_{n})=32m+\frac{729}{64}m(9n-5)$ with *m* and *n* as parameters. Second we see that this index rises with the rise in *m* and third shows the same tendency.

We conclude that all nine indices show the same behaviour.
